# Poroelastic behavior and water permeability of human skin at the nanoscale

**DOI:** 10.1093/pnasnexus/pgad240

**Published:** 2023-08-22

**Authors:** Ramin Oftadeh, Mojtaba Azadi, Mark Donovan, Jessica Langer, I-Chien Liao, Christine Ortiz, Alan J Grodzinsky, Gustavo S Luengo

**Affiliations:** Department of Biological Engineering, Massachusetts Institute of Technology, Cambridge, MA 02139, USA; Department of Biological Engineering, Massachusetts Institute of Technology, Cambridge, MA 02139, USA; School of Engineering, San Francisco State University, San Francisco, CA 94132, USA; L’OREAL Research and Innovation, Aulnay sous Bois, 93106, France; L'OREAL Research and Innovation, Clark, NJ 07066, USA; L'OREAL Research and Innovation, Clark, NJ 07066, USA; Department of Biological Engineering, Massachusetts Institute of Technology, Cambridge, MA 02139, USA; Department of Biological Engineering, Massachusetts Institute of Technology, Cambridge, MA 02139, USA; L’OREAL Research and Innovation, Aulnay sous Bois, 93106, France

## Abstract

Topical skin care products and hydrating compositions (moisturizers or injectable fillers) have been used for years to improve the appearance of, for example facial wrinkles, or to increase “plumpness”. Most of the studies have addressed these changes based on the overall mechanical changes associated with an increase in hydration state. However, little is known about the water mobility contribution to these changes as well as the consequences to the specific skin layers. This is important as the biophysical properties and the biochemical composition of normal stratum corneum, epithelium, and dermis vary tremendously from one another.

Our current studies and results reported here have focused on a novel approach (dynamic atomic force microscopy-based nanoindentation) to quantify biophysical characteristics of individual layers of ex vivo human skin. We have discovered that our new methods are highly sensitive to the mechanical properties of individual skin layers, as well as their hydration properties. Furthermore, our methods can assess the ability of these individual layers to respond to both compressive and shear deformations. In addition, since human skin is mechanically loaded over a wide range of deformation rates (frequencies), we studied the biophysical properties of skin over a wide frequency range. The poroelasticity model used helps to quantify the hydraulic permeability of the skin layers, providing an innovative method to evaluate and interpret the impact of hydrating compositions on water mobility of these different skin layers.

**Significance Statement**

Skin hydration is described as a beneficial healthy state of skin associated with optimal elasticity and radiance due to water uptake. We present a new experimental approach based on nanoindentation to quantify the biophysical properties of skin, especially hydration. The novelty of this approach is the use of a sponge-like description of skin tissue. Furthermore, we measure and model its biophysical properties and extract new data about the poroviscoelastic mechanical and hydraulic permeability of the different layers of human skin. Quantification of water mobility and hydraulic permeability is key to understanding skin's barrier function and the penetration of active substances. The perturbed equilibrium of the barrier function is the origin of certain skin dermatological conditions that together with environmental factors affect skin health.

## Introduction

Skin is a complex biological organ involved, among others, in processes associated with wound healing, skin hydration, and the regulation of body temperature. The surface area of the skin, which constitutes the largest tissue in our body, is estimated to be approximately 1.6 m^2^ (1) and is the body's first line of defense against microbes and harmful environments. In terms of material science, skin is a multiphase hierarchical structure, which results in a wide range of mechanical responses to changes in loading amplitudes and rates. Skin is generally considered to be a multilayer tissue consisting of the stratum corneum (SC), epidermis, and dermis.

The epidermis is an avascular tissue that consists of 95% keratinocytes. These cells proliferate, migrate and differentiate from lower layers to form four histologically discernible layers (2, 3). The outermost layer, called the s*tratum corneum* (SC), has a thickness of ~15–30 μm and is composed of a tight arrangement of flatten keratinized cells (corneocytes) embedded in a lipid matrix and connected through adhesion junctions called corneodesmosomes (4, 5). Due to its higher rigidity, SC's mechanical properties affect load transmission and deformation of the lower layers. SC is the first barrier against external agents and controls barrier function. Due to its water sensitive nature, a plasticization effect is easily observed for the SC, with the disruption of the intercellular lipid ordering being the target for many dermatological and cosmetic treatments (6). Changes in the water content of the SC can cause corneocytes to separate from each other, while still being supported by the force exerted by the corneodesmosomes. However, lateral in-plane interactions may contribute more to the separation between corneocytes compared to out-of-plane interlayer separation, as observed by Guo (4).

The epidermis consists primarily of viable keratinocytes arranged into several layers, including the basal layer, spinous layer, and stratum granulosum layer, where keratohyalin granules are present. The dermis, serving as the primary load-bearing component of the skin in tension, is considerably thicker than the epidermis, ranging from 15 to 40 times its thickness (1). The dermis consists of aggregated collagen bundles which account for ∼70% of its dry weight, elastic fibers, and an extrafibrillar matrix (1). The fibroblasts in the dermis produce a glycosaminoglycan matrix rich in hyaluronic acid, which contributes to skin hydration (7).

The out-of-plane mechanical properties of skin are highly nonlinear and anisotropic because of the morphology of each distinct layer and the heterogeneity within each layer. There are several in vivo approaches to characterize the overall mechanical properties of the skin, such as the Cutometer, which can measure local variations in elasticity, firmness, and hydration. However, the Cutometer may not be the most appropriate method to characterize specific mechanical properties of individual skin layers (8). In contrast, in vitro methods such as traction tests have the advantage of providing more accurate and controlled mechanical measurements of the skin. However, these methods may not fully represent the complexity of in vivo conditions and may not provide detailed information about individual skin layers. Recent studies using ultrasound elastography techniques (9, 10) are promising, though thickness resolution may be poor, especially regarding SC mechanical properties. Most studies suggest the need for a mechanical model of the skin to extract the relevant biomechanical properties (11).

Atomic force microscopy (AFM)-based methods have gained traction in recent years to evaluate micro and nanomechanical properties of whole skin and its individual layers (12–19). Most of these methods try to extract the contribution of each layer using models of diverse complexity. In all cases, the measured properties at the frequencies tested do not consider more than elastic or viscoelastic responses, without clear assessment of how water mobility might contribute to the response (19). To fit the experimental data, finite element (FE) models have proven to be robust and accurate approaches to estimating biomechanical properties of connective tissues, especially skin (20–26), with different constitutive equations derived for whole skin or each skin layer (22, 27).

Many previous studies have characterized the loading rate dependence of skin using “viscoelastic” models (e.g. “spring-dashpot” or prony series) to fit the data (13, 14, 28–33). While such models can be useful for fitting time dependence (e.g. stress relaxation), they do not represent the spatial depth dependence associated with water content of individual skin layers in terms of intrinsic, measurable rheological parameters such as the moduli and hydraulic permeability. The hydraulic permeability, in particular, is a biophysical parameter that quantifies the ability of water inside a skin layer to move “sideways” and vertically away from a mechanical stimulus (i.e. touch). The higher the hydraulic permeability, the faster the water can move away, increasing the rapidity of skin deformability and decreasing the ability of skin to maintain its turgor. This poroelastic effect is known to play an important role for example in tissues such as cartilage (34, 35) and has recently been computationally shown for human skin (36).

In this context, we hypothesized that extracting the “*poroviscoelastic*” properties of skin would be most important to understand skin hydration and our knowledge of skin biophysical properties, from a more dynamic perspective in which water mobility is better described. We recently developed a new high-bandwidth AFM-based rheology system for application to characterize the intrinsic poroelastic biophysical properties of cartilage (34, 35). This system and methodology, based on dynamic nanoindentation, were applied to human skin.

In this paper, we focused on a novel approach (dynamic AFM-based nanoindentation) to quantify biophysical characteristics of individual layers of ex vivo human skin. Since human skin is mechanically loaded over a wide range of deformation rates (frequencies), like all collagenous connective tissues, we studied the biophysical properties of skin over a wide frequency range. In addition, a poroelasticity model is developed to quantify the hydraulic permeability of the skin layers to evaluate and interpret the impact of hydrating compositions on water mobility of different skin layers.

Description of the SC in terms of poroelasticity goes beyond the classic “brick and mortar” model introducing the concept of water permeability with important consequences in skin diagnostics or the effect of hydrating compositions in skin.

## Materials and methods

### Poroviscoelastic finite element modeling

**Fig. 1.**
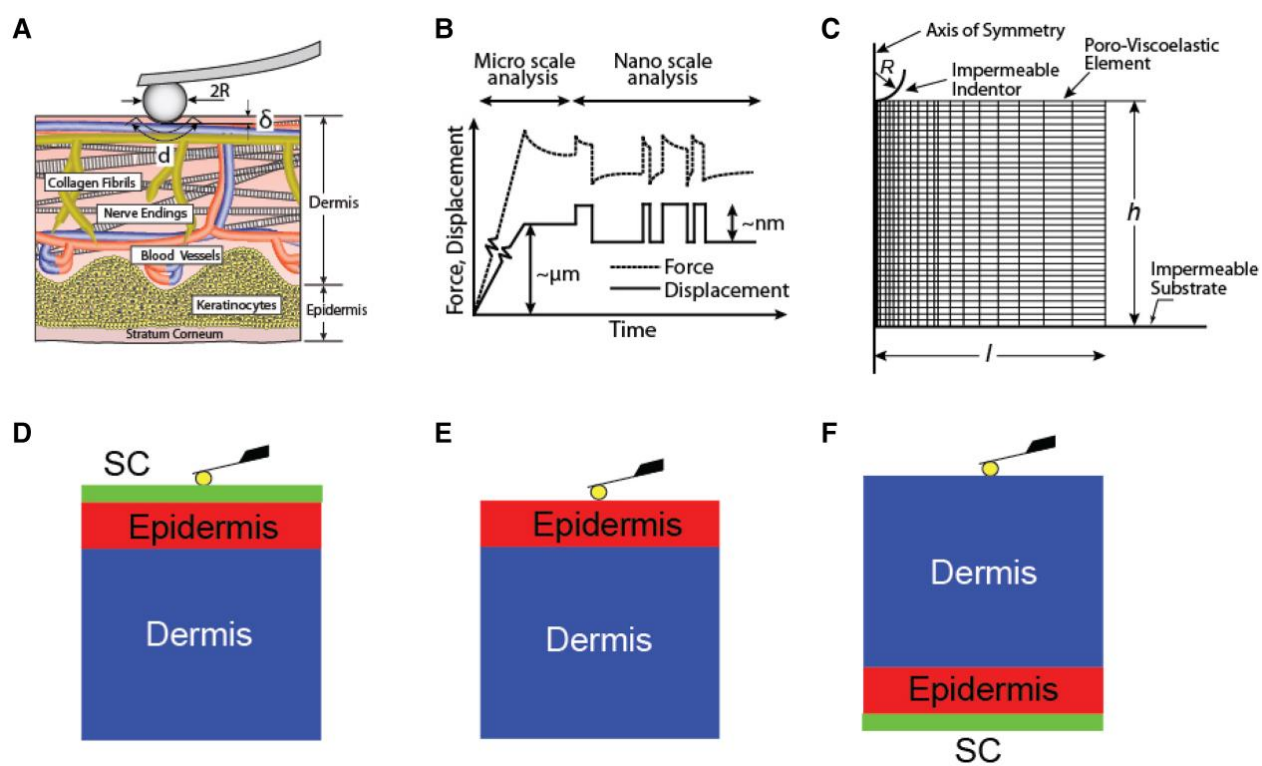
(A) Schematic of a skin sample tested using high-bandwidth AFM nanorheology with inset highlighting the presence of cells, blood vessels, nerves, and randomly oriented collagen fibril network within the dermal matrix. (B) The displacement profile applied to specimens composed of an initial ramp-and-hold displacement at the microscale followed by a random binary sequence of steps having a displacement amplitude of ∼2–15 nm. (C) Schematic of two-dimensional axisymmetric finite element (FE) model highlighting the poroviscoelastic element, impermeable indentor, axis of symmetry, and impermeable substrate. (D–F) AFM nanoindentation of the layers of human skin including (D) the stratum corneum (SC), epidermis (E), and dermis (F).

We developed a new method for predicting the mechanical properties of skin tissue using AFM nanoindentation. This involved developing a FE model that incorporated fibril reinforcement and poroviscoelasticity. We used the spherical probe tip of radius *R*, which made contact with the tissue at a length of *d*, to measure the properties of the tissue. The current model incorporates viscoelasticity (20), and it is an advancement of the model used earlier for poroelasticity of cartilage (34, 37, 38). The skin samples collagen network has a random orientation in the plane perpendicular to the probe tip face. To represent such structures, we used a two-dimensional axisymmetric model (34, 38) (Figure 1A–F).

In our experimental setup, we considered the AFM probe as a rigid entity, constructed from polystyrene material. This choice was justified by the probe's high modulus (*E* ∼3 GPa), which surpasses the compression modulus of the skin samples (*E* < 1 MPa). To replicate the experimental conditions accurately, we assumed that both the probe tip and substrate surface exhibit impermeability to fluid flow, while the contact between the indenter and substrate is frictionless (34). For simulation purposes, we set the pore pressure to zero at the tissue surface, excluding the interface with the probe tip, as well as at the lateral surfaces of the tissue. This approach aimed to simulate the drainage of interstitial fluid from the tissue at those specific surfaces.

The model employed in this study incorporated two main components as follows: an isotropic nonfibrillar matrix, representing the non-collagenous extracellular matrix (ECM), and a fibrillar network, specifically collagen, which accounted for the anisotropic nature of the skin across all three layers. The construction of the model was executed within the ABAQUS, utilizing its capabilities for soil mechanics. Within the model, the poroelastic tissue mechanical properties encompassed several parameters as follows: the hydraulic permeability (*k*), the elastic modulus of the nonfibrillar matrix ($E_{m}$), the Poisson's ratio (ν), and the fibrillar modulus ($E_{f}$). The poroelastic relaxation time ($T_{p}$) was found to be proportionate to the square of the characteristic contact length at the interface between the probe and tissue ($d^{2}$), while inversely proportional to the product of hydraulic permeability (*k*) and the elastic modulus of the nonfibrillar matrix ($E_{m}$) (39). Additionally, we incorporated the viscoelastic properties by utilizing a standard three-element linear solid model. The model included the same nonfibrillar modulus ($E_{m}$) in parallel with a Maxwell solid, consisting of a modulus ($E_{v}$) in series with a dashpot. Viscoelastic properties are represented by a standard three-element linear solid consisting of the same nonfibrillar modulus $E_{m}$ in parallel with a Maxwell solid (i.e. a modulus $E_{v}$ in series with dashpot *η*, such that the viscoelastic relaxation time is $T_{v}=\eta$/$E_{v}$), where *η* is the material coefficient of viscosity. These adjustments and considerations allowed for the representation of the mechanical behavior of the skin tissue, encompassing both poroelastic and viscoelastic properties within the model framework.

We formulated a poroviscoelastic constitutive model incorporating solid and fluid phases. Drawing inspiration from the work of Wilson et al. (40), our approach involved assuming the porous solid phase to be completely saturated with water. This solid phase comprised a nonfibrillar matrix, representing interfibrillar linkers and proteoglycans, along with a fibrillar network composed of collagen. As a result, the material's overall stress ($\sigma_{\mathrm{total}}$) can be expressed as follows:


(1) $$\sigma_{\mathrm{total}}=\sigma_{m}+\sigma_{f}-pI.$$

In this context, the stress within the nonfibrillar matrix is denoted as $\sigma_{m}$, while the stress within the fibrillar network is represented by $\sigma_{f}$. The fluid pressure is indicated by *p*, and the unity tensor is denoted as *I*.

In this three-element viscoelastic solid, we can express the stress–strain relationship of the nonfibrillar matrix as follows (20):


(2) $$\sigma_{m}(t)+\frac{\eta }{E_{v}}\frac{d\sigma_{m}(t)}{dt}=E_{m}\varepsilon_{m}(t)+\left(\frac{(E_{m}+E_{v})\eta }{E_{v}}\right)\frac{d\varepsilon_{m}(t)}{dt}.$$

Here, *σ* represents stress, while *ε* denotes strain. The stress within the fibril matrix is presumed to exhibit a linear dependence on strain and is solely activated when the fibrils are subjected to tension. This characteristic aligns with the recognized tension–compression nonlinearity commonly observed in various connective tissues characterized by fibrous composition (41):


(3) $$\{\begin{array}{l} \sigma_{f}=E_{f}\varepsilon_{f}\;\;\;\;\;\;\;\;\;\;\;\mathrm{for}\;\;\;\;\;\;\;\;\;\varepsilon_{f}>0\\ \sigma_{f}=0\;\;\;\;\;\;\;\;\;\;\;\;\;\;\;\mathrm{for}\;\;\;\;\;\;\;\;\;\varepsilon_{f}<0\;\;\;\; \end{array}.$$

Incorporating the fibrillar network into the model involved discretizing the network into spring elements. The stiffness of these spring elements was determined by equating the strain energy of the network to the strain energy of the discretized springs (38).

### Sample preparation

We tested both macroscopic and nanoindentation methodologies and established protocols using four skin donor samples obtained from L’Oréal R&I. We found that the measured mechanical properties of fresh vs. thawed skin tissue could not be distinguished (see Fig. S1 and Table S1 in Supplementary material). In addition, and very importantly, we developed a protocol to give reliable and repeatable data using AFM-based nanoindentation.

This study utilized normal human skin samples acquired from surgical remnants of abdominoplasty tissue provided by healthy volunteers (Zenbio Inc.), in compliance with the ethical guidelines outlined in the Declaration of Helsinki. The participants provided written informed consent, and the relevant documentation was retained by the attending dermatologist. We received the samples de-identified, with only limited information such as age, sex, and anatomical site disclosed. The authors were not involved in the collection of the samples. The 14 human skin donor samples were ∼1.5 cm × 1.5 cm pathogen-free samples obtained from female donors (listed in Table 1) provided frozen in saline and maintained in this state in our lab until testing.

**Table 1.** Skin samples’ specification.

Sample ID#	Location	Age	Gender	Ethnicity
1	Abdominal	62	Female	CC
2	Abdominal	68	Female	CC
3	Abdominal	64	Female	CC
4	Abdominal	60	Female	CC
5	Abdominal	66	Female	CC
6	Abdominal	59	Female	CC
7	Abdominal	70	Female	CC
8	Abdominal	60	Female	CC
9	Abdominal	70	Female	CC
10	Abdominal	63	Female	AA
11	Abdominal	60	Female	AA
12	Abdominal	65	Female	CC
13	Abdominal	56	Female	CC
14	Abdominal	69	Female	CC

CC, Caucasian ethnicity; AA, Afro-American ethnicity.

Since these 14 samples (average age 63.7) represented a more homogeneous population than initially anticipated, we proceeded to test all 14 using our AFM-based wide bandwidth rheology approach, to focus on comparing the properties of the SC with those of the epidermis and dermis, in order to gain insights into their respective variations.

The skin samples were sliced horizontally and using a tissue slicer, the skin was sliced in a direction parallel to the unfrozen surface. The resulting slices were 0.5 mm thick, and their diameter was 9 mm. Our measurements show that the thickness was not significantly different among the skin samples. AFM calibration for nanoindentation was performed in saline buffer at room temperature. Samples were then mounted in the AFM and maintained under fully hydrated conditions with phosphate buffered saline (PBS) throughout testing. Tests were performed after environmental equilibrium was reached. In Fig. 1A, a schematic representation of the skin samples utilized in the high-bandwidth AFM nanorheology experiments is depicted. The inset emphasizes the presence of various components such as cellular structures, nerve components, blood vessels, and the dermal matrix containing a randomly oriented network of collagen fibrils.

The tissue samples were preserved in a PBS solution supplemented with protease inhibitors to maintain their physiological ionic strength. Mechanical testing was conducted within 2 h after thawing the samples. To secure the samples onto the custom stage, a minimal layer of cyanoacrylate glue was used, ensuring their stability. Throughout the experiments, the samples were consistently hydrated with PBS to prevent dehydration. To mitigate any undesired boundary effects arising from glue or tissue edges, the indentations were strategically executed in proximity to the tissue center. Each sample underwent indentations in multiple locations, typically ranging from 10 to 15 sites, with a minimum of 5 to 10 indentations conducted per location.

AFM tests were first performed on full thickness skin (Fig. 1D) with the SC face-up. SC was then removed via tape stripping (using scotch tape with 15–20 repeated tape strips) (14), and nanoindentation tests were performed again on the same sample (Fig. 1E). Then, a second sample from the same donor specimen was mounted on the AFM setup with the dermis face-up; nanoindentation was thereby performed on the dermis layer (Fig. 1F). Since the nanoindentation displacement amplitudes (∼2–15 nm) is much less than the thickness of each skin layer, it is assumed that the properties of the specific layer on top dominate measurements in the configuration of Fig. 1D to 1F.

### Assessment of loading conditions and data processing

In order to obtain the dynamic complex modulus of the skin across a broad frequency spectrum (ranging from 1 Hz to 10 kHz), a proprietary high-frequency rheology system integrated with a commercially available atomic force microscope (MFP-3D, Asylum Research, Santa Barbara, CA) was employed (39, 42, 43). At the core of the system is a secondary piezo, referred to as the primary component. Distinguished from the primary piezo, which serves as the z-piezo in the commercial AFM, the secondary piezo is deliberately designed to be compact (measuring 2 × 2 × 2 mm). This compact size allows for an expanded range of attainable frequencies by pushing the resonance frequency of the combined piezo system toward higher values. For our experiments, we utilized colloidal probe tips made of polystyrene obtained from Polysciences (Warrington, PA). These probe tips exhibited a range of radii, approximately ranging from 2.5 to 25 μm. To mount the probe tips, we employed tipless cantilevers with a nominal spring constant of approximately 7.4 N/m from Budget Sensors (Sofia, Bulgaria). The precise spring constants of all tips were determined through direct measurements utilizing the thermal calibration technique (44). Drawing from prior research, we opted for a specific loading protocol for indentation. This approach entailed an initial pre-indentation phase with a ramp-and-hold pattern at the microscale, denoted as δ (as depicted in Fig. 1A), ranging approximately from 0.5 to 4 μm. Subsequently, we implemented random binary sequence (RBS) displacements at the nanoscale, as shown in Fig. 1B. These displacements were characterized by dynamic amplitudes ranging between 2 and 8 nm. To execute this protocol, we relied on established techniques and custom software tools (43). Given that the dynamic displacement amplitudes applied ranged between 2 and 8 nm, significantly smaller in magnitude compared to both the sample size and the radius of the probe tip, our hypothesis was that the resultant tissue strains would be infinitesimal in nature. Considering this, we posited that employing a linear poroviscoelastic theory (as discussed in Section 2.1.2) would offer a suitable framework for comparison with the experimental findings presented in this study.

For this investigation, the measurement sampling rate was established at *f_s_* = 100 kHz, ensuring a comprehensive capture of data. The duration of the time series was set to *T* = 30 s, allowing for an adequate representation of the signal characteristics. To filter out higher frequency noise, a low pass filter was implemented with a cutoff frequency of *f_c_* = 1 Hz. Employing a discrete Fourier transform, the fundamental frequency components of the force signal $F_{osc}$ and the displacement signal δ were extracted for further analysis (39). The dynamic complex indentation modulus, with its magnitude and phase, was derived from the measured force and applied displacement data. This computation was carried out using the built-in “etfe” function in MATLAB, which utilizes the Fourier transform method to extract the frequency-dependent characteristics of the modulus.

### Statistical analyses

Statistical analysis was conducted by performing multiple indentations at 10 to 15 different locations within each skin layer. At each location, three indentations were performed, and the resulting data were averaged. To represent the overall trend, the mean values along with their corresponding 95% confidence intervals were calculated. This analysis was carried out on a total of 14 skin layer samples (*n* = 14).

## Results and discussion

**Fig. 2.**
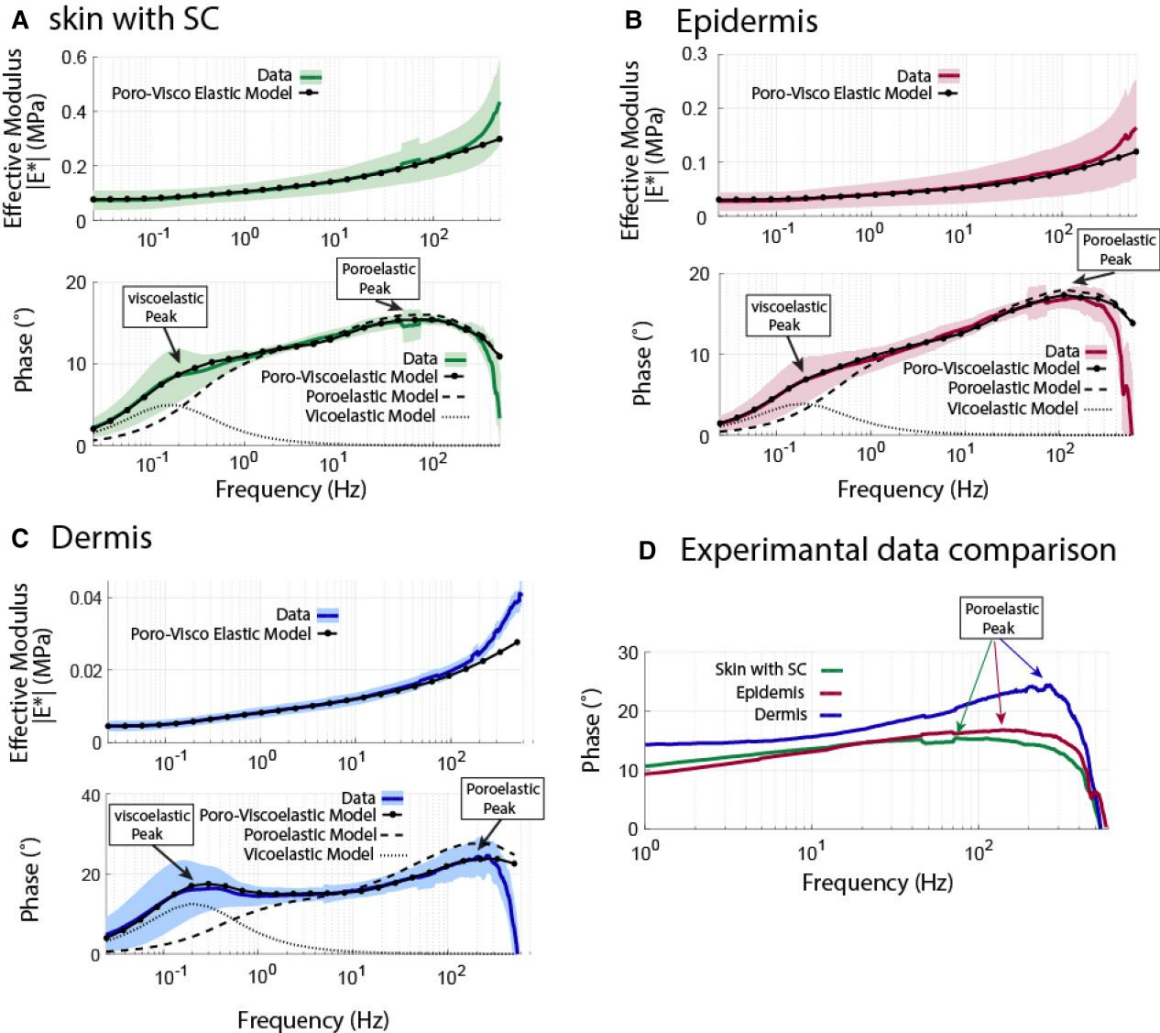
Magnitude and phase of the dynamic complex modulus of the (A) stratum corneum (SC), (B) epidermis, and (C) dermis of a representative human skin sample, measured by AFM-based nanoindentation. Shaded areas correspond to ±95% confidence intervals. The dermis (C) shows the most pronounced separation of the poroelastic and viscoelastic peaks in the phase angle. Solid lines are data, dotted, and dashed lines are models based on viscoelasticity alone, poroelasticity alone, and the combined fibril-reinforced poroviscoelastic FE model. D shows the poroelastic peak shift among skin layers.

We quantified the magnitude, represented as $\left|E^{*}\right|$, and the phase angle, denoted as *ϕ*, of the dynamic modulus each of the three layers of the 14 human skin samples, where *ϕ* represents the phase difference between the force and the applied displacement. A representative example of each layer for one skin sample is shown in Fig. 2. $\left|E^{*}\right|$ for all three cases of SC, epidermis, and dermis decreases to an asymptotic low frequency Young's modulus of *E_m_* ∼90 kPa, 48 kPa, and 27 kPa, respectively (Fig. 2A to 2C). We immediately note, therefore, that the dermis has a significantly lower low frequency (equilibrium) Young's modulus compared to the epidermis which, in turn, is much lower than the SC. At high frequencies (∼500 Hz), the magnitude of $E^{*}$ increased approximately threefold to *E_H_* ∼230 kPa, 164 kPa, and 86 kPa, respectively; thus, the dermis presented the weakest resistance to compression even at high frequencies.

Significantly, the dominant frequency at which the phase angle reaches its maximum, coupled with the frequency-dependent behavior of the magnitude of $E^{*}$, holds crucial significance in deducing the intrinsic poroelastic characteristics of a tissue through nanoindentation. Furthermore, it allows for the exploration of the intricate relationship between these nanoscale poroelastic properties and the molecular structure as well as the composition of the ECM. For the SC, the phase angle *ϕ* exhibited a peak at the frequency of *f*_peak_ = 110 Hz, diminishing toward zero at both lower and higher frequencies. Comparable patterns were observed for the epidermis and dermis, although with upward shifts in the peak frequency to *f*_peak_ = 150 and 200 Hz for the epidermis and dermis, respectively.

The phenomenon of self-stiffening, characterized by the augmentation of the dynamic modulus $\left|E^{*}\right|$ as the loading frequency increases, serves as a protective mechanism for skin cells and their surrounding pericellular matrix. This mechanism involves an elevation in the stiffness of the tissue ECM in response to dynamic loads, thereby safeguarding the cells.

Energy dissipation in the skin, associated with the tangent of the phase angle ϕ, involves the dispersal of impact energy through poroelastic dissipation, thus mitigating potential damage. At the nanoscale, different skin layers demonstrate self-stiffening behavior, characterized by an increase in the dynamic modulus from the range of 27–90 kPa to 86–230 kPa across the frequency range of 1–500 Hz. A distinct trend of self-stiffening is observed in conjunction with an optimal frequency corresponding to the peak phase angle. For the SC, this peak frequency is approximately ∼110 Hz, while for the epidermis and dermis, it shifts to around 150 and 200 Hz, respectively.

The shift in the peak phase angle with frequency for SC, versus epidermis versus dermis (as observed in Fig. 4), indicates that hydraulic permeability (*k*) increases from SC to epidermis to dermis; therefore, water flow upon compression of skin is highest in dermis.

Combined fibril-reinforced poroviscoelastic FE model is shown for both magnitude and phase (Fig. 2). Individual poro and viscoelastic models cannot predict the full-frequency response spectrum for all the layers, and the superposition of both models is needed for full cover of the frequency response spectrum. Two peaks are visible on the phase angle figures; one peak represents the viscoelastic response, while the other peak corresponds to the poroelastic behavior. The viscoelastic peak occurs in lower frequency ranges while poroelastic peak occurs in high-frequency ranges (11). As we go deeper into skin layers (from SC to dermis), the poroelastic peak shifts to the right which implies higher hydraulic permeability (Fig. 2D); while viscoelastic peak does not shift noticeably. The phase angle in the dermis (Fig. 2C) exhibits the clearest distinction between the poroelastic and viscoelastic peaks.

In order to validate the poroelastic nature of the prominent peak in the phase angle, we investigated the relationship between the dynamic modulus and characteristic length scale. By taking into account the influence of characteristic length scale on poroelastic dissipation and the scale independence of intrinsic viscoelastic dissipation, we investigated the correlation between the peak frequency of the phase angle, denoted as *f*_peak_, and the reciprocal of the contact area (1/*d*^2^) formed between the AFM probe tip and the underlying skin layer.

**Fig. 3.**
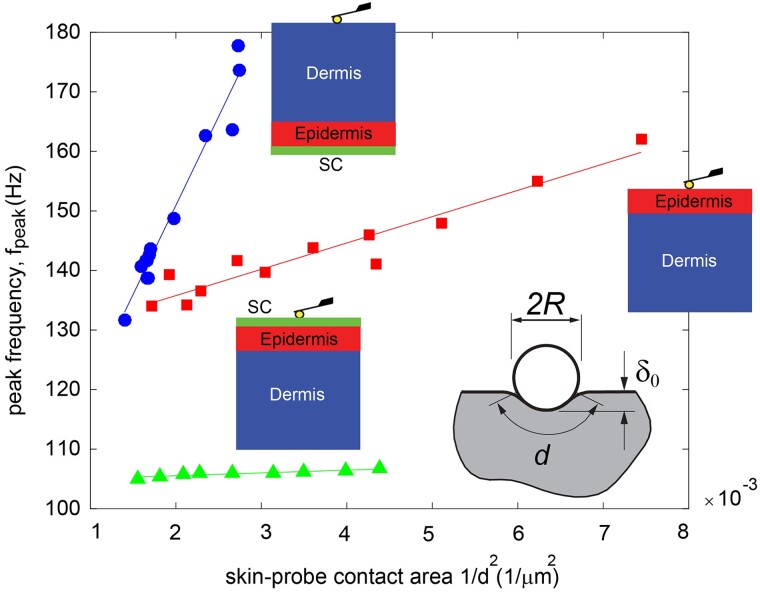
The characteristic length scale dependence of the dynamic modulus was studied by examining the relationship between the peak frequency of the phase angle, $f_{\;peak}$, and the inverse of the contact area (proportional to 1/*d*^2^) between the AFM probe tip and underlying skin layer. Most importantly, in the higher frequency poroelastic regime of testing, as identified in Fig. 2D above, the linear relationship between peak frequency and inverse contact area (1/*d*^2^) observed in this skin sample is a hallmark of the behavior of a poroelastic material, which clearly distinguishes this regime of behavior from that of a viscoelastic material.

The characteristic frequency, *f*_peak_, can be defined based on the principles of linear poroelastic theory as follows:


(4) $$f_{\mathrm{peak}}\;\propto \frac{k\;E_{L}}{d^{2}},$$

where $E_{L}$ is the low frequency modulus (45, 46). Based on this equation, the linear relationship between peak frequency and inverse contact area (1/*d*^2^) (Fig. 3) observed in a skin sample is a hallmark of the behavior from that of a viscoelastic material. Furthermore, the comparison of slopes reveals that the ($E_{L}\;$*k*) product is higher for the dermis in contrast to the epidermis and SC layers. On the other hand, magnitude figures in Fig. 2 reveal that low frequency modulus $\left(E_{L}\right)$ of dermis is lower compared to epidermis and SC. Therefore, to compensate for the lower $E_{L}$ in the $E_{L}k$ product, the hydraulic permeability of dermis should be greater compared to epidermis and *k* of epidermis should be higher compared to SC.

**Fig. 4.**
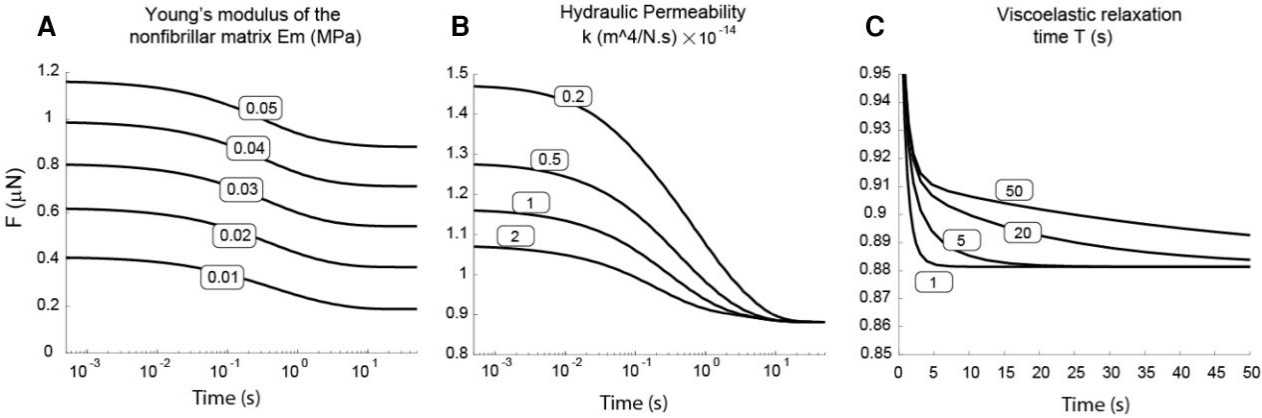
The effect of varying model parameter values on the force-displacement response during indentation of the skin model. (A) Increasing the nonfibrillar modulus $E_{m}$ while holding all other parameters constant increases the force required to achieve the same final displacement over the entire duration of the indentation test. (B) Increasing the hydraulic permeability *k* while holding all other parameters constant decreases the force at the beginning of relaxation but has no effect on the force at the final time when stress relaxation is reached. (C) Increasing the viscoelastic relaxation time *T* while holding all other parameters constant increases the force at longer times during the relaxation period but has no effect on early time points.

As mentioned in the Materials and methods section, prior to applying RBS displacement, a preliminary ramp-and-hold displacement with a magnitude of approximately ∼1–2 μm was imposed on the tissue. This set of experiments enables us to further explore the mechanics of the tissue at the microscale. Furthermore, the obtained results were compared with existing findings in the literature.

From a modeling point of view, the important mechano-physical properties of the model have been changed to explore the effect of each one on micromechanics response. Figure 4(A) illustrates an increase in the elastic modulus of the nonfibrillar matrix, while keeping all other parameters constant in the model (i.e. $k=1e-14\;(m^{4}N/s)$, $E_{f}=0.1\;\mathrm{MPa}$, $G_{v}/G_{m}=0.05\;$, T=5s). Increasing $E_{m}$ increases the force caused by indentation to the same final displacement by a constant amount over the entire duration from 10^−1^ to 50 s. Therefore, tuning $E_{m}$ can be used to move the entire curve by a constant amount of force. In Fig. 4(B), increasing hydraulic permeability while maintaining the constancy of all other parameters within the model (i.e. $E_{m}=0.05\;\mathrm{MPa}$, $E_{f}=0.1\;\mathrm{MPa}$, $G_{v}/G_{m}=0.05$, T=5s) decreases the force at the beginning of relaxation but has no effect on the force at the final time when the force relaxation is reached. The hydraulic permeability can be used to tune the beginning of force relaxation curve. While maintaining the constancy of all other parameters within the model, Fig. 4(C) demonstrates the impact of increasing the viscoelastic relaxation time. (i.e. $k=1e-14\;(m^{4}N/s)$, $E_{m}=0.05\;\mathrm{MPa}$, $E_{f}=0.1\;\mathrm{MPa}$, $G_{v}/G_{m}=0.05$) increases the force at longer times during force relaxation period while has no effect on early time points. The *T* can be used to tune the end of the force relaxation curve.

**Fig. 5.**
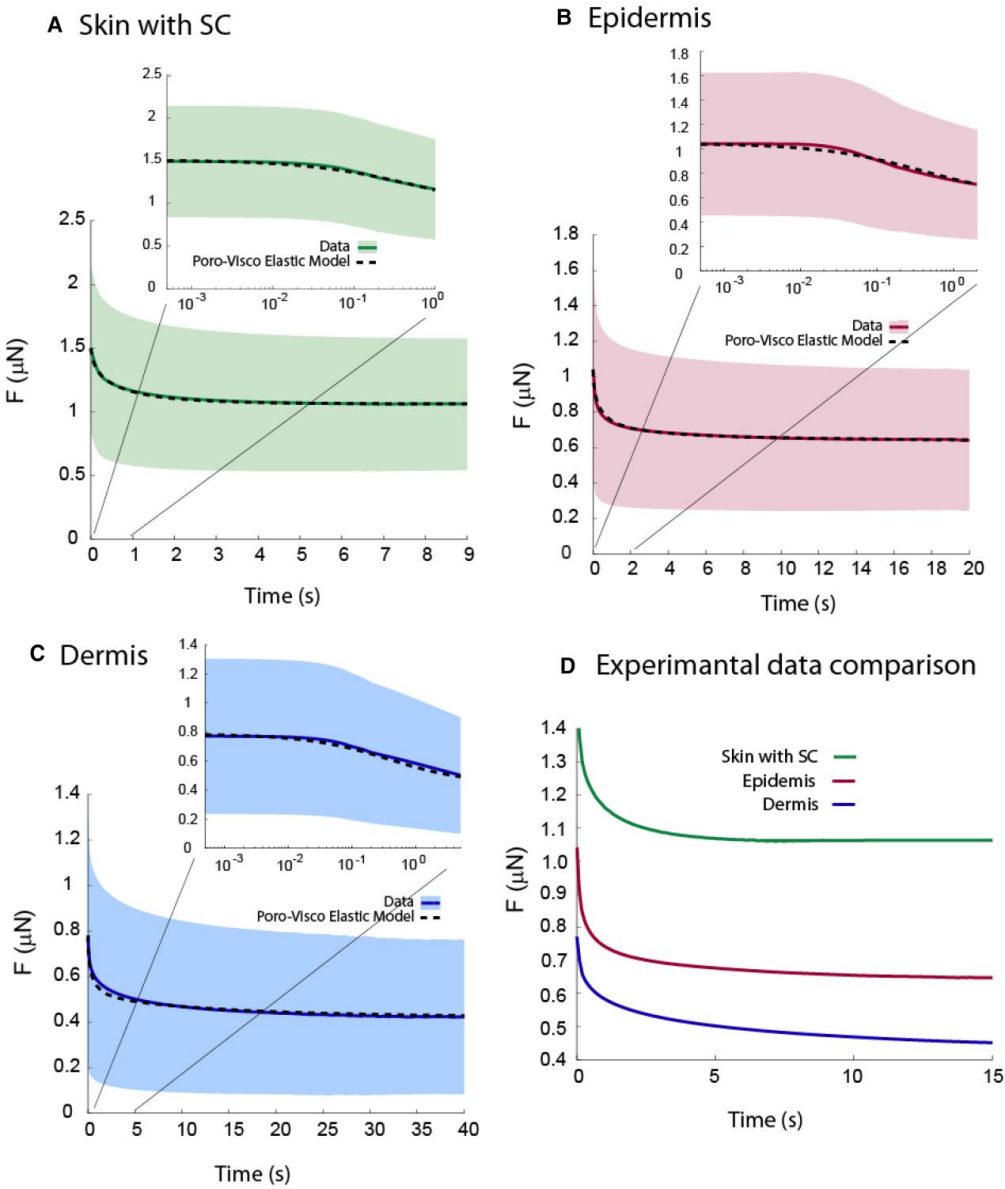
Force relaxation of the (A) stratum corneum (SC), (B) epidermis, and (C) dermis of a representative human skin sample, measured by AFM-based microscale indentation. Shaded areas correspond to ±95% confidence intervals. For more clear visualization, the beginning of the relaxation period for each skin layer is magnified and shown as an inset at the top of each curve. (D) Comparison of stress relaxation shows a higher residual force and slower relaxation in the SC compared to the faster relaxation to a lower residual force in the epidermis and dermis, given higher nonfibrillar elastic modulus of SC compared to other skin layers.

Figure 5 shows the force relaxation of three skin layers for a representative skin sample. For each layer, the beginning of the relaxation period is magnified and shown as an inset on the top of each curve. The finite element fit for each curve is shown as a dotted line. The parametric study analysis shown in Fig. 4 has been used to fit the poroviscoelastic FE model to the data. First, low frequency modulus $E_{m}$ tuned to fit the portion of the curve where the relaxation is reached. Then, viscoelastic relaxation time (*T*) and hydraulic permeability (*k*) are tuned to fit the force at the beginning and end of the relaxation. Figure 5(D) shows the relaxation comparison of individual skin layers. SC has the highest residual force and fastest relaxation, whereas dermis has the lowest residual force and slowest relaxation compared to other skin layers. The high residual force of SC can be justified given its higher nonfibrillar elastic modulus.

**Fig. 6.**
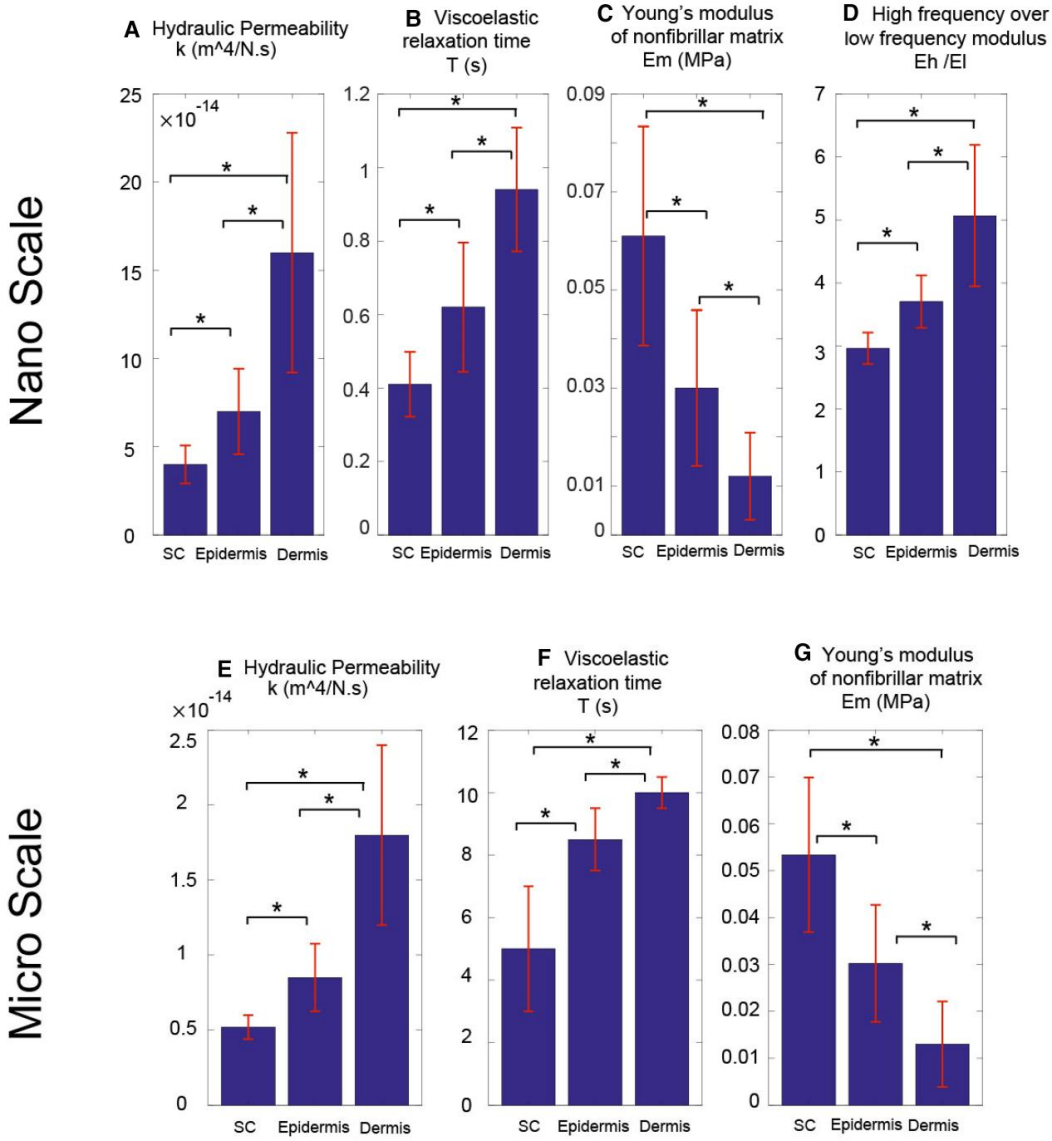
Material properties of human skin obtained by fitting our fibril-reinforced poroviscoelastic FE model to data obtained from AFM-based nanoindentation measurements of the dynamic complex modulus on all 14 human skin samples (including the sample associated with Fig. 2), for nanoindentation analysis (top). In addition, material parameters calculated from microscale analysis of the same 14 samples (bottom, including the data of Fig. 5). Values are mean ± 95% confidence interval. *P < 0.05 for all cases.

The poroviscoelastic FE model was employed to fit the data acquired from three distinct skin layers, namely the SC, epidermis, and dermis. By conducting this analysis, the poroviscoelastic material properties for all 14 human skin samples were estimated. Figure 6 shows the mean values ± 95% confidence interval for the hydraulic permeability (*k*), viscoelastic relaxation time (*T*), nonfibrillar matrix Young's modulus ($E_{m}$), and high frequency over low frequency modulus ratio (*E_h_*/*E_l_*) and averages over all 14 samples. The results are shown for nanoscale and microscale analyses.

For both microscale and nanoscale, the hydraulic permeability is highest in the dermis and least in the SC (Figs. 6A and E). These results show the ability of dermis to hold and move the water more easily compared to other skin layers. The hydraulic permeability is an order of magnitude higher at the nanoscale compared to microscale for all three layers. This difference can be interpreted as the ability of water to move more easily at the nanoscale compared to microscale. We therefore believe that our current method can be used to detect the effect of skin additives (e.g. hyaluronan) on the properties of different skin layers.

Figures 6B and F also show that viscoelastic relaxation time is higher in the dermis compared to other skin layers. It can be explained by the dermis having a rich collagen network, which contributes to the viscoelasticity of the dermis. Viscoelastic relaxation times at the microscale are an order of magnitude longer compared to those at the nanoscale. This can be interpreted as a higher amount of strain at the microscale compared to nanoscale, which causes the force to relax more slowly at the microscale.

Figures 6C and G also shows that the nonfibrillar matrix Young's modulus is generally lowest in the dermis and highest in the SC. This can be explained by the many layers of keratin-filled corneocytes in the SC, which make this layer stiffer. In addition, this shows that as cells migrate from the deeper layers in the epidermis to the SC, they become stiffer. The order of magnitude of $E_{m}$ for both microscale and nanoscale is the same for all the layers. Since we are indenting the same place and material for both microscale and nanoscale, the amount of stiffness does not change between scales.

Figure 6D illustrates the self-stiffening ratio, which reveals a trend from the dermis to both the epidermis and SC in terms of the differrence in mechanical stiffness at higher versus lower frequencies. The bigger this ratio, the stiffer the tissue is at higher loading rates relative to lower loading rates, because of the restriction to fluid flow at high rates. These observed changes indicate a reduction in the effectiveness of fluid–solid frictional interactions to enhance intra-tissue hydrostatic pressurization in the upper skin layers. Moreover, the findings highlight the significant role of the dermis in providing protection during high loading rate activities and resisting impact-related injuries.

Generally, a wide range of Young's moduli from the order of kPa to GPa have been reported for the skin (47). This is mainly due to the method of testing (14, 48), skin nonlinearity (47, 49), strain rate and range (30), and tissue type (21, 29). In terms of AFM methods which include nano and microindentation of the tissue, the reported Young modulus ranges from order of kPa to MPa (12–18, 20). The main reasons for this wide range can be attributed to the tissue type (13, 18, 20), having pre-stretching the tissue (17), touching the areas with a large amount of collagen network (for dermis) (18), and probe tip size (13, 50). Therefore, the Young's moduli we have reported have been measured qualitatively to compare the elasticity of the different skin layers.

Using AFM, Achterberg et al. (16) determined the Young's modulus of dermis samples from 20 human female donors and found that it varies from 0.1 to 10 kPa, depending on the specific body area and the corresponding dermal layer. For microscale analysis, we found the Young's modulus of dermis to be between 3.9 kPa and 22.1 kPa, which is consistent with their results. The small discrepancy between the results could be because of (or due to) the experimental measurements themselves (e.g. the AFM force-displacement curves used for the Hertz model versus our full-frequency scans [Fig. 2] and our time-relaxation curves [Fig. 5]), as well as the different methods that have been used to measure the Young's modulus. In their study, Young's modulus was determined by fitting the contact region of the force-indentation curves using a standard Hertz model. The Hertz model assumes isotropic, homogeneous, and linear elastic material behavior, whereas in our analysis, we employed an anisotropic, linear poroviscoelastic FE model (Fig. 1C) to fit the force relaxation curves (Fig. 5).

Based on the Hertz model modified for flat indenters, Crichton et al. (13) quantified the elastic properties of mouse full thickness skin using fabricated probes. They found that the elastic modulus for 10 μm probe size is 1.31 ± 0.80 MPa which is in contrast to our finding for elastic modulus of SC (0.053 ± 0.016) which represents full thickness skin. This discrepancy can be caused by the tissue type and method of measuring Young's modulus. They used skin from the back of mice ears and used flat indenters for nanoindentation.

In terms of hydraulic permeability of the skin, most studies have been focused on skin barrier physical properties (51–56) and many noninvasive methods have been developed for this purpose (57). Hydraulic permeabilities reported in the literature are reported for SC and epidermis. Using transepidermal water loss measurements (56), absorption/desorption experiments (58), numerical simulations (59), and MRI measurements (60), the hydraulic permeability of epidermis reported between 1e^−14^ to 1e^−15^ m^4^/Ns which is in line with our results hydraulic permeability of epidermis (0.85e^−14^ ± 0.23e^−14^ m^4^/Ns).

In terms of physical barrier, our results show that the SC has the lowest hydraulic permeability, which indicates that it is the strongest barrier to dehydration, protein loss, and plasma component leakage from the tissue. On the other hand, dermis has the highest hydraulic permeability, which makes it suitable to allow water flow inside the tissue to keep the tissue hydrated and its high hydraulic permeability makes it suitable for injectable fillers and drugs to propagate throughout the tissue.

## Conclusion

To our knowledge, the results presented here provide the first definitive evidence of the poroelastic behavior of skin. While several older papers in the literature have hypothesized the possibility of such behavior based on previous poroelastic and mixture theories for hydrated tissues and biomaterials, we do not know of previous experimental evidence documenting global poroelastic behavior of skin and identifying distinct differences in such properties within the different layers of skin. In addition, this behavior provided a quantitative description of the essential importance of water mobility using a more detailed description of skin's structure.

We believe that the experimental methodologies implemented in this study offer a distinctive approach to quantifying the effects of specific additives to the hydration, turgor, swelling, and deformation behavior of distinct skin layers, potentially useful for quantifying the effects of hyaluronan or other possible treatments.

Our results to date suggest that the observed poroelastic behavior is especially and most evident in the dermis, which is consistent with an increase in water mobility (7, 61). Our results suggest that the dermis is most susceptible to additive treatment, and that changes in material property behavior of the dermis would likely alter the behavior of the full thickness skin as a result. Still this approach can have an important impact in the other layers and in particular SC. Furthermore, our newly developed methods would allow for the changes of poroviscoelastic behavior in ageing and dry skin in addition to skin conditions such as atopic dermatitis.

## Supplementary Material

pgad240_Supplementary_Data

## Data Availability

All the necessary data required to evaluate the conclusions drawn in this study are presented within the manuscript.
