# Supplementary material for: Poroelastic behavior and water permeability of human skin at the nanoscale
Source: PNAS Nexus. 2023 Aug 22;2(8):pgad240. doi: 10.1093/pnasnexus/pgad240 (PMC10443659; doi:10.1093/pnasnexus/pgad240)
Supplement: pgad240_Supplementary_Data [file pgad240_supplementary_data.pdf]

Elasticity of fresh samples versus frozen samples

Before conducting the main experiments on human samples, a preliminary study was conducted to find the effect of freezing on the mechanics of the skin samples. 4 samples were tested. Mechanical indentation, removing SC (Stratum Coronium) and analysis of the data were performed based on the protocols explained in the main manuscript.

Result: No significant difference was observed in the nanoscale elasticity of fresh samples without SC versus frozen samples without SC. While freezing had no effect on elasticity between fresh and frozen samples, removing the SC of fresh samples, itself, resulted in significantly softer tissue at both the micro- and nanoscale.

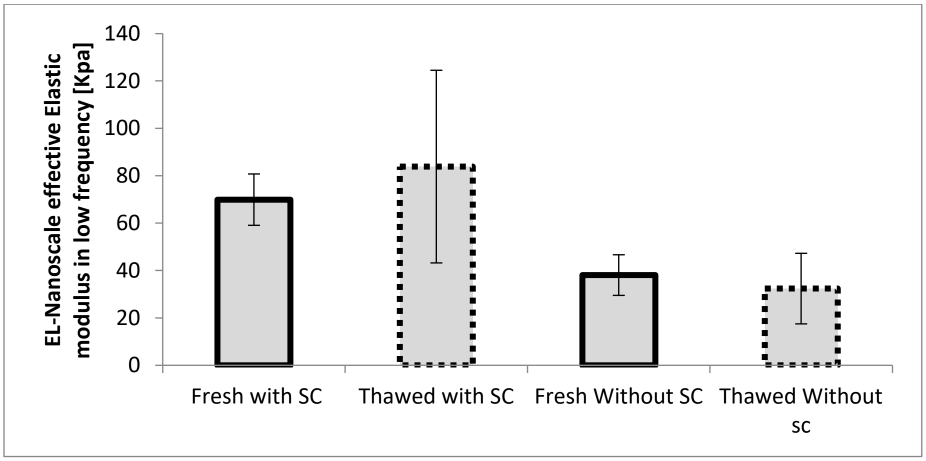

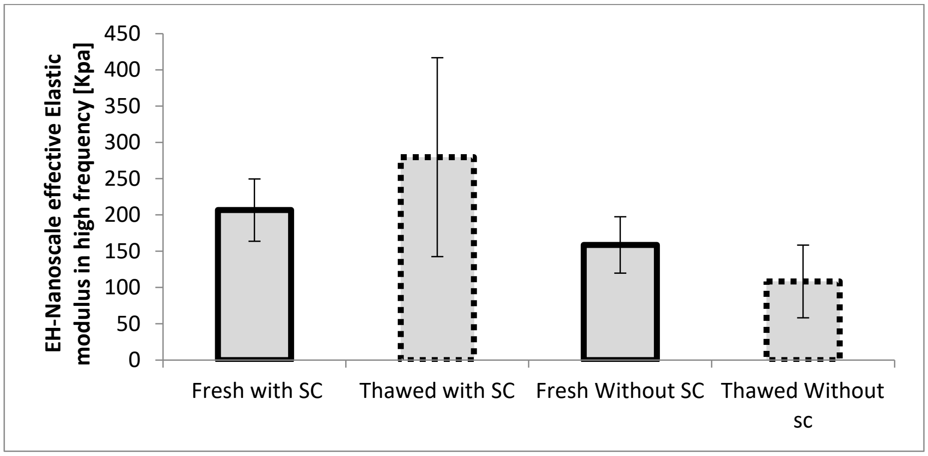

Figure S1. Nanoscale Elasticity of fresh and thawed skin tissues with stratum coronium and without stratum coronium measured at low frequency [up] and high frequency [Bottom]. The low frequency limit of the dynamic complex modulus (EL-Nanoscale; i.e., measured at the nano scale) is equivalent to the equilibrium elastic modulus. The low frequency Elastic Modulus (EL) and high frequency Elastic Modulus (EH) are the left-most and right-most data points of the Magnitude of the dynamic complex modulus shown in Figure 2A of the manuscript. The dynamic complex modulus curves of fresh and thawed were similar.

Table S1. Data related to the Nanoscale Elasticity of fresh and thawed skin tissues with stratum coronium and without stratum coronium measured at low frequency [up] and high frequency [Bottom].

	**Sample number**	**Number of Indentation test on each sample**	**EL**	**STD**	**P-Value**	**EH**	**STD**	**P-Value**
Fresh **with** SC	4	>10	69.92	10.82	0.378	206.75	42.92	0.321
Thawed **with** SC	4		83.85	40.66		279.73	137.18	
Fresh **Without** SC	4		38.08	8.56	0.3774	158.70	38.95	0.229
Thawed **Without** SC	4		32.40	14.90		108.25	50.13	
